# In-depth analysis of the safety of CAR-T cell therapy for solid tumors

**DOI:** 10.3389/fimmu.2025.1548979

**Published:** 2025-02-24

**Authors:** Jiayi Dong, Jiexiong Wu, Ye Jin, Zhu Zheng, Ting Su, Lijuan Shao, Jiaxin Bei, Size Chen

**Affiliations:** ^1^ Department of Immuno-Oncology, The First Affiliated Hospital of Guangdong Pharmaceutical University, Guangzhou, China; ^2^ Key Laboratory of Monitoring Adverse Reactions Associated with Chimeric Antigen Receptor T-Cell Therapy, Guangdong Higher Education Institutions, Guangdong Pharmaceutical University, Guangzhou, China; ^3^ Guangdong Provincial Engineering Research Center for Precision Medicine in Esophageal Cancer, The First Affiliated Hospital of Guangdong Pharmaceutical University, Guangzhou, China; ^4^ Key Laboratory of Cancer Immunotherapy, Guangdong Higher Education Institutions, Guangdong Pharmaceutical University, Guangzhou, China; ^5^ School of Clinical Medicine, Guangdong Pharmaceutical University, Guangzhou, China

**Keywords:** CAR-T cells, tumor immunity, solid tumors, tumor therapy, adverse reactions

## Abstract

In recent years, the rapid progress in oncology, immunology, and molecular biology has dramatically advanced cancer immunotherapy, particularly CAR-T cell therapy. This innovative approach involves engineering a patient’s T cells to express receptors that specifically target tumor antigens, enhancing their ability to identify and eliminate cancer cells. However, the effectiveness of CAR-T therapy in solid tumors is often hampered by the challenging tumor microenvironment (TME). The complex TME includes dense stroma that obstructs T cell infiltration, abnormal blood vessel structures leading to hypoxia, and an acidic pH, all of which hinder CAR-T cell function. Additionally, the presence of immunosuppressive factors in the TME reduces the efficacy of CAR-T cells, making successful targeting of tumors more difficult. The safety of CAR-T therapy has gained interest, especially CAR-T therapy has shown considerable effectiveness in various cancers, with notable results in multiple myeloma and hepatocellular carcinoma, among others. Nonetheless, CAR-T cell therapy is associated with several adverse reactions primarily driven by heightened levels of proinflammatory cytokines. These reactions include cytokine release syndrome (CRS), neurotoxicity (CANS), and organ toxicity, often leading to serious complications. CRS, characterized by systemic inflammation due to cytokine release, can escalate to severe organ dysfunction. It typically occurs within the first week post-infusion, correlating with CAR-T cell expansion and often presents with fever and hypotension. Meanwhile, CANS encompasses neurological issues ranging from mild symptoms to severe seizures, possibly exacerbated by CRS. Organ toxicity can also arise from CAR-T therapy, with potential damage affecting the gastrointestinal tract, kidneys, liver, and lungs, often tied to shared antigens found in both tumor and healthy tissues. Moreover, long-term effects like cytokine-associated hematotoxicity (CAHT) and secondary malignancies represent significant concerns that could affect the patient’s quality of life post-treatment. The long-term adverse effects and challenges in treating solid tumors underscore the need for ongoing research. Strategies to improve CAR-T cell efficacy, minimize adverse reactions, and enhance patient safety are critical. Future explorations could include designing CAR-T cells to better navigate the TME, identifying specific target antigen profiles to minimize off-target damage, and developing adjunct therapies to mitigate cytokine-related toxicity. Continued monitoring for long-term effects will also be paramount in improving patient outcomes and maintaining their quality of life. Overall, while CAR-T therapy holds great promise, it must be administered with careful consideration of potential side effects and rigorous management strategies to ensure patient safety and treatment efficacy.

## 1 Introduction

In recent years, the rapid advancements in oncology, immunology, and molecular biology have also fueled the rapid development of cancer immunotherapy (1, 2). CAR-T cell therapy is a cutting-edge cellular immunotherapy technique that utilizes gene engineering to construct and express receptors that specifically recognize tumor antigens on the patient’s own T cells, thereby equipping these T cells with “super soldier”-like precision in identification and efficient killing capabilities to attack specific tumor cells. In clinical practice, CAR-T cell therapy for solid tumors, such as glioblastoma multiforme (GBM), encounters challenges due to the complexity of the tumor microenvironment (TME), potentially resulting in complex adverse reactions. In HER2 CAR-T therapy, delayed fever occurred in two patients (3). In autologous HER2 CAR-T cell treatment for patients with advanced sarcoma, dose-limiting toxicity was observed, resulting in grade 3-4 CRS, which can be life-threatening (4). Studies on EGFRvIII-directed CAR-T therapy for GBM have shown that patients’ baseline characteristics and changes in EGFRvIII percentage are associated with the risk of adverse reactions (5) (NCT02209376). In solid tumors, stromal cells contribute to a dense fibrotic TME that restricts the movement and infiltration of CAR-T cells (6), while the abnormal vascular architecture can lead to tissue hypoxia, affecting the expression of key molecules necessary for T cell adhesion and further hindering their infiltration (7, 8). The acidic pH often found in these tumors can adversely impact CAR-T cell function, making effective targeting more difficult (9). Additionally, the TME harbors various soluble immunosuppressive factors, such as IDO, IL-10, and TGF-β, which dampen the activity and functionality of CAR-T cells, reducing their anti-tumor effectiveness (10–12). Furthermore, the heterogeneity of tumor antigen expression allows some cells to evade detection, and tumor cells can adopt escape mechanisms, including downregulating tumor-specific antigens or upregulating checkpoint molecules, to protect themselves from CAR-T cell attacks (13). Finally, the lack of necessary adhesion molecules within the TME limits CAR-T cell movement and accumulation (14). Together, these interrelated factors present considerable challenges for the success of CAR-T cell therapies in treating solid tumors.This study aims to explore the safety of CAR-T cell therapy for solid tumors, aiming to benefit more patients. Since the groundbreaking success of CAR-T cell therapy in curing Emily Whitehead, the first child with relapsed refractory leukemia in 2012, this treatment has attracted significant global interest. CAR-T therapy has shown remarkable effectiveness in various cancers. For example, BCMA CAR-T therapy has achieved impressive results in advanced IgGλ multiple myeloma, with some patients reaching complete remission within two weeks (15). CAR-T therapies targeting GPRC5D have also proven effective for patients who relapse after BCMA treatment (16). Additionally, CStone Pharmaceuticals’ GPC3-targeted CAR-T therapy has yielded notable outcomes in advanced hepatocellular carcinoma (HCC), with two patients remaining tumor-free for over seven years (17). In a Stanford trial, an innovative GD2 CAR-T therapy benefited patients with diffuse midline glioma (DMG), as nine out of eleven patients experienced improved neurological function, and one patient even achieved complete tumor disappearance, remaining cancer-free four years after treatment (18).

## 2 Manuscript

### 2.1 Mechanisms of adverse reactions associated with CAR-T cell therapy

**Figure 1:**
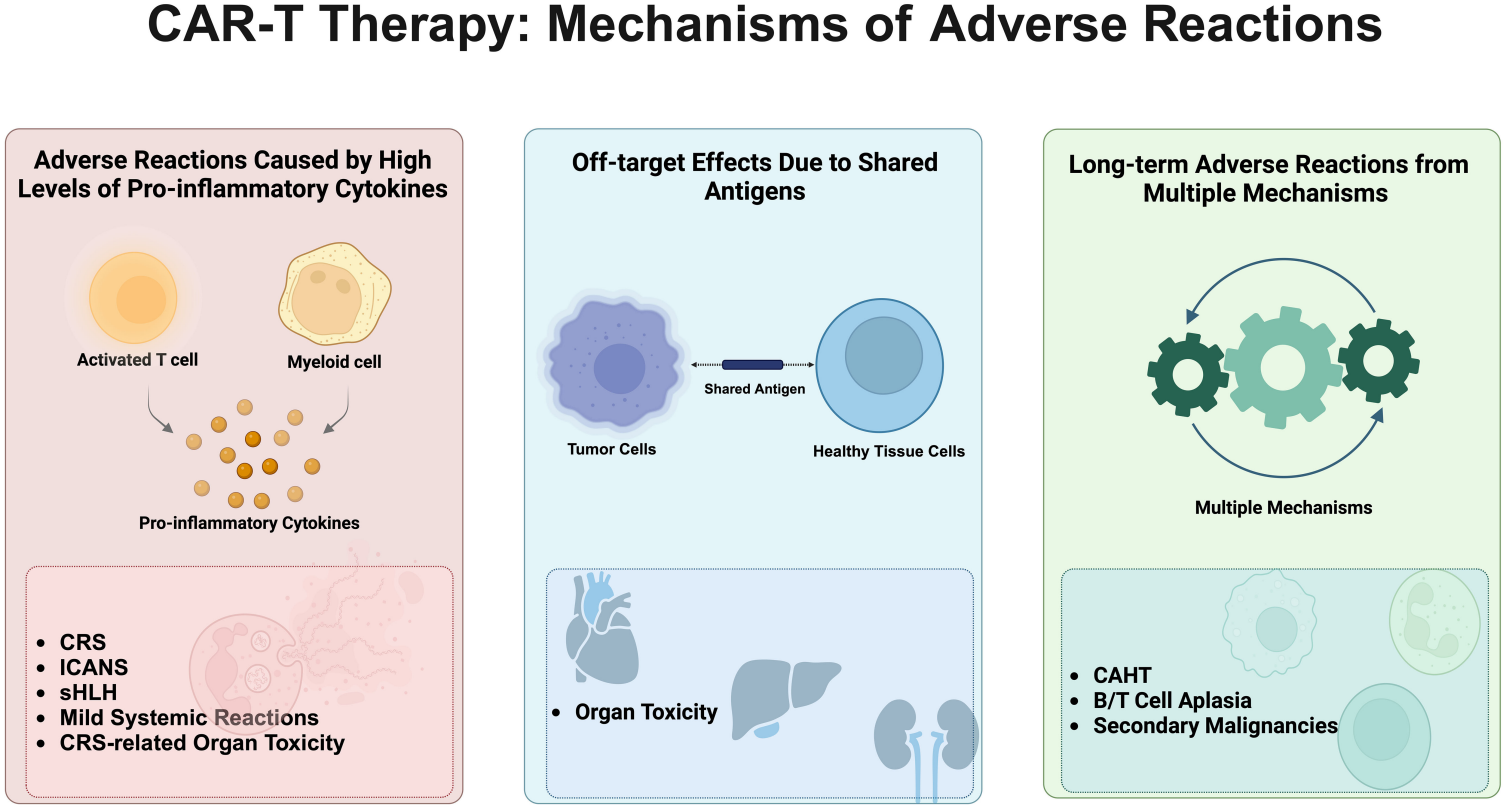
Mechanisms of adverse reactions associated with CAR-T cell therapy. This figure vividly illustrates the primary adverse reaction mechanisms triggered by CAR-T cell therapy, described sequentially from left to right. Initially, it details reactions induced by high levels of pro-inflammatory cytokines, including Cytokine Release Syndrome (CRS), Immune Effector Cell-Associated Neurotoxicity Syndrome (ICANS), Secondary Hemophagocytic Lymphohistiocytosis (sHLH), mild systemic reactions, and CRS-related organ toxicity. Following this, the diagram shows off-target effects caused by shared antigens between tumor and healthy cells, leading to significant organ toxicity. Lastly, long-term adverse effects arise from a complex interplay of mechanisms, encompassing cell-associated hematotoxicity (CAHT), B/T cell aplasia, and secondary malignancies. The comprehensive depiction of these mechanisms not only reveals the potential risks associated with CAR-T therapy but also emphasizes the importance of meticulous monitoring and management of patients undergoing this treatment.

The adverse reactions associated with CAR-T cell therapy are caused by multiple mechanisms. Firstly, they are primarily induced by high levels of proinflammatory cytokines secreted by activated T cells and myeloid cells (19). These adverse reactions include cytokine release syndrome (CRS) (20), cell-associated neurotoxicity syndrome (CANS) (19), secondary hemophagocytic lymphohistiocytosis (sHLH) (21), systemic reactions with mild symptoms as the main manifestation, and CRS-related organ toxicity. Secondly, due to the presence of shared antigens between tumor and healthy tissues, off-target effects can lead to organ toxicity, which is relatively common in solid tumor clinical trials (22). Thirdly, long-term adverse reactions caused by the combined action of multiple mechanisms include cell-associated haematotoxicity (CAHT), B/T cell aplasia, and secondary primary malignancies (SPMs) (23) (**Figure 1**).

#### 2.1.1 cytokine release syndrome

CRS is a severe adverse reaction in CAR-T cell therapy (24), representing a severe systemic inflammatory response syndrome triggered by the activation of immune cells and the massive release of cytokines such as tumor necrosis factor-α (TNF-α), interleukins (ILs), and interferons (IFNs) (20, 25).Severe CRS can lead to organ dysfunction. CRS typically occurs in the first week after infusion, coinciding with the peak expansion phase of CAR-T cells, and its severity correlates with the activity and number of these cells. Activated CAR-T cells release a large amount of cytokines, further exacerbating CRS, while the lysis and apoptosis of tumor cells also contribute to the occurrence of CRS (19, 26). IL-6 exhibits a biphasic peak phenomenon after CAR-T cell therapy, which predicts life-threatening infections (27) and triggers a series of clinical symptoms such as fever, hypoxia, and hypotension. CRS grading is based on the severity of symptoms, ranging from Grade 1 fever to Grade 4 severe hypotension and hypoxia (28, 29). Treatment for CRS includes medications (e.g., immunosuppressants, cytokine inhibitors) (19, 30, 31) and supportive measures (e.g., oxygen therapy, fluid replacement) (32). Tocilizumab is an FDA-approved therapy for CRS but is ineffective against CANS (29). In severe cases, therapeutic plasma exchange or hemofiltration may be required (33). Previous studies have warned that high-dose CAR-T therapy may lead to fatal Grade 4 CRS in patients (34). Therefore, it is particularly important to strictly control the preparation process of CAR-T cells, conduct comprehensive assessments before infusion, and closely monitor patients during treatment (19). This includes continuous observation of patients during and after infusion to promptly detect symptoms of CRS and rapidly intervene (35). Despite the potential severe consequences of CRS, moderate CRS responses are actually associated with better tumor response rates and patient survival, highlighting the importance of balancing risks and benefits during treatment (36).

#### 2.1.2 cell-associated neurotoxicity syndrome

While CAR-T cell therapy demonstrates great potential in cancer treatment, it is accompanied by a severe complication that cannot be ignored: CANS. CANS is a series of neurological abnormalities caused by the activation of endogenous or exogenous T cells and other immune effector cells after CAR-T cell infusion, with diverse clinical manifestations and varying severity (19, 37). Symptoms can range from mild speech disorders to potentially fatal seizures (38, 39). Studies have confirmed that epileptiform discharges are associated with the rapid progression to higher grades of CANS within 24 hours (40). CANS in CAR-T cell therapy refers to a series of neurological abnormalities caused by the activation or involvement of endogenous or exogenous T cells and other immune effector cells in patients after CAR-T cell infusion.

CANS typically appears on days 4 to 5 after CAR-T cell reinfusion and sometimes occurs simultaneously with or subsequent to CRS, which may exacerbate the development of CANS (41). CRS, as a strong immune response, is considered a potential trigger or contributing factor for CANS, with higher grades of CRS often accompanied by higher grades of CANS. In extreme cases, CRS combined with neurotoxicity may lead to a rapid deterioration of the patient’s condition, accompanied by blood abnormalities, liver and kidney function damage, coagulation disorders, and even intracranial hemorrhage (42). It may also trigger secondary hemophagocytic lymphohistiocytosis (HLH), a life-threatening complication that requires prompt intervention to avoid rapid death (21, 43). Patients with HLH have significantly higher levels of IL-10, which helps distinguish HLH from severe CRS. Serum lactate dehydrogenase levels and fibrinogen levels can predict the development of CAR-T-cell-induced HLH (carHLH), providing valuable insights for the prevention and treatment of carHLH (44). In the treatment of CAR-T cell-induced HLH, a variety of medications and therapies constitute the primary strategies. Tocilizumab, an IL-6 receptor antagonist, is commonly used to treat CAR-T-related HLH and CRS, with most patients receiving this treatment to alleviate symptoms (45). Steroids, particularly dexamethasone, are also widely employed to control inflammation and modulate immune responses, with treatment duration tailored to individual responses (46). Furthermore, Anakinra, an IL-1 receptor antagonist, has demonstrated potential in the treatment of CAR-T-related HLH, aiming to further reduce inflammatory responses (47, 48). For patients with severe primary or CAR-T-related HLH refractory to other treatments, chemotherapy drugs such as etoposide and cyclophosphamide may be considered as therapeutic options (49). For patients with persistently elevated IL-6 levels, siltuximab provides another pathway to control inflammation (50). Basiliximab, an IL-2 receptor-blocking antibody, has also been trialed in some cases of CAR-T-related HLH (51). Additionally, anti-thymocyte globulin and the JAK2 inhibitor ruxolitinib have been explored for regulating the immune system and reducing inflammation (52, 53). In terms of supportive care, continuous venovenous hemodiafiltration (CVVH) plays a crucial role in correcting electrolyte imbalances, acid-base disturbances, and fluid management, aiding in renal function recovery and preventing severe complications (54). Early initiation of CVVH can mitigate the clinical symptoms of CAR-T-related HLH and shorten the duration of immunosuppressive therapy (55). In specific circumstances, the CytoSorb extracorporeal cytokine adsorption device is also used to adsorb and remove excess cytokines from the body, thereby reducing inflammatory responses (56).

Although the exact mechanism of CANS is not fully elucidated, several hypotheses have been proposed. These include the possibility that cytokines released by CAR-T cells cross the blood-brain barrier, triggering inflammation and neuronal damage in the central nervous system (19), direct entry of CAR-T cells into cerebrospinal fluid causing neuronal damage (57), and alterations in immune status induced by CAR-T therapy that may exacerbate neurological damage (58). Additionally, CAR-T cells activate tumor-associated macrophages, leading to increased secretion of the pleiotropic cytokine IL-1. The widely expressed IL-1 receptor is responsible for pro-inflammatory signaling, and studies have found that IL-1 plays a crucial role in the pathogenesis of CANS.

A clinical study (NCT03692429) of CAR-T therapy CYAD-101 for unresectable metastatic colorectal cancer (mCRC) resulted in two patient deaths due to CANS-related adverse events, ultimately leading to the termination of the project. A systematic review indicated that most CAR-T-related deaths are associated with blood-brain barrier disruption, central nervous system cell damage, and infiltrating T cells (59).

With the rapid development of artificial intelligence and machine learning technologies, we are capable of constructing predictive models based on big data to accurately identify patients who are more susceptible to immune cell-associated neurotoxicity syndrome (ICANS) (60, 61). This advancement provides physicians with potent tools for pretreatment risk assessment, enabling them to adopt targeted preventive measures. By extensively collecting and analyzing clinical data from patients undergoing CAR-T therapy, encompassing dimensions such as age, gender, underlying disease status, pretreatment tumor burden, and CAR-T cell infusion dosage, we can train highly efficient machine learning models to predict the occurrence of ICANS. Furthermore, delving into biomarkers associated with the pathogenesis of ICANS, such as specific cytokine level changes and gene expression profiles, will further enhance the accuracy of these predictive models (62). Serving as early warning signals, these biomarkers can assist physicians in swiftly responding to effectively mitigate ICANS symptoms (63). Currently, the primary clinical approach to managing severe toxicities associated with CAR-T therapy is systemic corticosteroid treatment. However, we should not rest on our laurels but actively explore other effective treatments, such as intrathecal corticosteroid injection and anti-IL-6 antibody therapy, and strive to develop novel drugs to more effectively alleviate or prevent neurotoxicity (47). In terms of CAR-T cell therapy, optimizing the specific CAR structure to reduce its attack on normal tissues and regulating the proliferation and activation state of CAR-T cells can lower the risk of overreaction (64). Simultaneously, it is essential to actively explore safer and more effective immunocellular therapies, such as CAR-NK cell therapy and CIK cell therapy, to provide patients with more options. Ultimately, formulating individualized treatment strategies based on patients’ specific conditions and ICANS risk factors is crucial. For high-risk patients, more aggressive preventive measures and rigorous monitoring should be implemented; for patients who have already developed ICANS, careful selection of a treatment plan based on the severity of their symptoms and pathophysiological mechanisms is necessary to achieve optimal therapeutic outcomes (65).

#### 2.1.3 Systemic reaction

##### 2.1.3.1 Flu-like symptoms

Fever is one of the most common adverse reactions after CAR-T cell therapy, typically occurring within 24 hours of cell infusion. It is noteworthy that isolated fever is usually not considered a direct manifestation of CANS or CRS. For this adverse reaction, non-steroidal anti-inflammatory drugs (NSAIDs) are widely used in clinical treatment, and most patients experience a rapid normalization of body temperature after treatment. Further research has shown that the fever response induced by CAR-T cell therapy is not affected by treatment specificity or CAR target, and fever is common in patients with gastric cancer and lung cancer undergoing single-target CAR-T cell therapy (66, 67). However, if fever occurs 21 days or more after CAR-T cell infusion, it may indicate the possibility of delayed-onset lung toxicity, which may be closely related to upregulated PD-L1 expression and toxicity to normal lung tissue (68). The underlying mechanisms of fever mainly involve the release of cytokines and nonspecific activation of immune responses, but it is important to note that the absence of a fever response cannot be directly used as a basis for judging the absence of therapeutic effect.

Similar to fever, headache as a single symptom has a low correlation with ICANS and CRS. CRS-induced neurological symptoms tend to appear earlier and present with more extensive encephalopathy symptoms, lacking a direct correlation with headache. In clinical practice of CAR-T therapy for solid tumors, headaches are more common after treatment in patients with intracranial tumors, which may be related to the influence of the primary tumor site. Additionally, some patients may experience mild flu-like symptoms, including headache (69), such as joint pain and myalgia, which are more pronounced with high-dose CAR-T cell infusion and tend to worsen progressively with increasing doses. Notably, high-dose CAR-T cell infusion can induce grade 3 headaches, and some patients may not respond well to oral analgesics (70). Furthermore, occasional occurrences of non-dose-limiting headaches have been reported in patients with seminoma and colon cancer (71, 72), which may be closely related to cytokine-driven immune responses.

Mild fatigue is one of the common adverse reactions in CAR-T cell therapy for solid tumors, with its specific mechanism not fully elucidated. A phase I study targeting malignant pleural mesothelioma, ovarian cancer, and pancreatic ductal adenocarcinoma showed that nearly half of the subjects experienced fatigue after CAR-T cell therapy (73). Although there was no significant correlation between the occurrence of fatigue and dose or lymphocyte depletion rate, it still requires attention from clinicians during treatment. As of now, there have been no reports of death due to fatigue in solid tumor patients after CAR-T cell infusion.

##### 2.1.3.2 Allergic reaction

Although the overall risk of allergy in CAR-T cell therapy for solid tumors is relatively low, specific patient populations still face the potential threat of allergic reactions. Reports have shown that patients with a history of multiple allergies to anti-PD-1 antibodies and platinum-based drugs are prone to anaphylactic shock after CAR-T treatment, but fortunately, most patients can recover after timely and effective therapeutic intervention (66). Additionally, immune reactions induced by murine-derived single-chain variable fragments (scFv) in CARs are also an important cause of allergic reactions (73). It is noteworthy that when treating pleural mesothelioma with CAR-T cells, special vigilance is required regarding the risk of allergic reactions and acute respiratory distress syndrome (ARDS). Therefore, it is recommended that such patients receive the minimum recommended dose of cell therapy and undergo close monitoring within 48 hours after treatment (74). Future research should further focus on patients’ allergy histories, explore optimal therapeutic doses and mechanisms suitable for allergic populations, and minimize the risk of allergic reactions.

#### 2.1.4 Organ toxicity

CRS, as a major inducing factor, can cause organ toxicity, with gastrointestinal toxicity, renal toxicity, pulmonary toxicity, and hepatic toxicity occurring less frequently than CRS and CANS. Currently, there are clear clinical diagnostic indicators and guidelines for these toxicities. Abnormal elevations of inflammatory cytokines such as IL-6, VWF, Ang-2, and TNF-α, as well as target cross-reactivity of CAR-T cells to actin, can lead to cardiovascular toxicity. CRS is also one of the inducing factors for skin toxicity, manifesting clinically as urticaria, vesicular ulcerations, and oral mucositis. The understanding of CRS-induced immunosuppression contributing to skin toxicity is still insufficient, potentially due to skin infections in patients. Currently, there are no diagnostic and treatment guidelines for skin toxicity. Patients with severe CRS are more prone to neutropenia, which is closely related to infectious complications and the occurrence of late-onset hematological toxicity (75). Besides high levels of pro-inflammatory cytokines associated with CRS, various organ toxicities are also related to tissue-targeting effects. The following paragraphs will provide a detailed review of these aspects.

### 2.2 Adverse reactions related to shared antigens between tumor and healthy tissues

**Figure 2:**
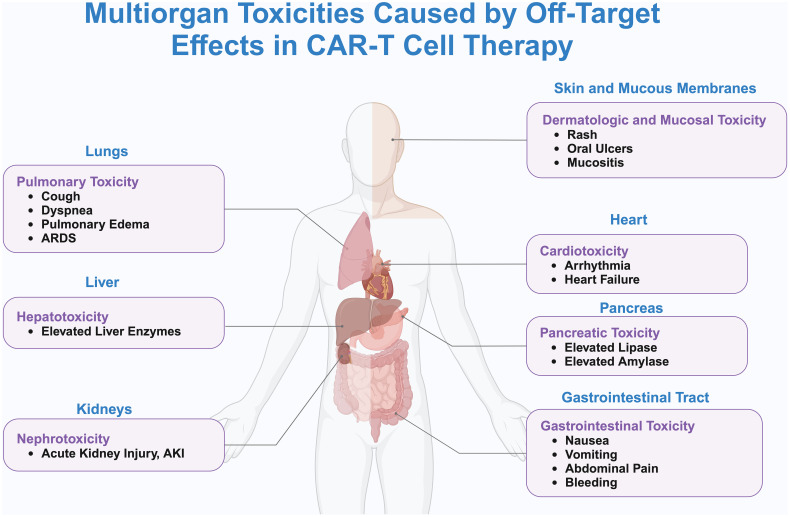
Systemic toxicity and off-target effects caused by CAR-T cell therapy. This figure delineates the systemic toxicities associated with off-target effects of CAR-T cell therapy, as visualized through a human body silhouette that maps the principal organs affected and their specific adverse reactions. Detailed are the pulmonary toxicities in the lungs, including cough, dyspnea, pulmonary edema, and ARDS; hepatotoxicity in the liver, evidenced by elevated liver enzymes; nephrotoxicity in the kidneys, marked by AKI; cardiotoxicity in the heart, manifesting as arrhythmias and heart failure; pancreatic toxicity in the pancreas, characterized by increased levels of lipase and amylase; gastrointestinal toxicity in the digestive tract, presenting as nausea, vomiting, abdominal pain, and bleeding; and dermatologic and mucosal toxicity, involving rashes, oral ulcers, and mucositis. Lines connecting these adverse reactions to CAR-T cells underscore the systemic nature of these off-target effects. The illustration emphasizes the critical need for rigorous monitoring and management of patients receiving CAR-T therapy to effectively mitigate the risks of multiorgan toxicity.

In anti-cancer treatment, the tumor antigens that need to be targeted should ideally only be expressed on tumor cells or at very low levels on normal cells, and these tumor antigens are referred to as tumor-specific antigens (TSAs) (76). However, TSAs are rare, and most of the antigens currently used in CAR-T therapy are tumor-associated antigens (TAAs), which are expressed at low levels on other healthy cells. CAR-T cells injected into the body can kill both tumor cells and normal cells that express the target antigen, a phenomenon known as tissue-targeting effect. The tissue-targeting effect can cause severe side effects and even death (42). “On-target/off-tumor” (OTOT) toxicity may cause damage to healthy cells and organs (**Figure 2**).

#### 2.2.1 Cardiovascular toxicity

Cardiovascular complications induced by CAR-T cell therapy have been reported infrequently in the treatment of solid tumors, with reports of cardiac toxicity mainly focusing on clinical studies in gastric cancer (66) and colorectal cancer (72). Furthermore, CRS is associated with cardiac toxicity, and early management of CRS can mitigate cardiac toxicity (77). High-risk patients should undergo cardiac assessment and, in cases of suspected cardiovascular adverse events, electrocardiogram, echocardiogram, and biomarker testing should be performed (78–82).

#### 2.2.2 Gastrointestinal toxicity

Nausea and vomiting during CAR-T therapy are mostly related to preconditioning medications (3, 83), rather than the CAR-T cells themselves. Gastrointestinal bleeding is more common in tumors of the digestive system and is associated with local inflammation, often being reversible (3). In one case, a patient experienced rapid tumor regression accompanied by Grade 4 gastrointestinal bleeding shortly after CAR-T cell infusion (66). Abdominal pain is relatively common in digestive system tumors (66, 73, 78, 84), and the influence of preconditioning medications should also be considered in such cases (72). Therefore, gastrointestinal adverse reactions during CAR-T cell therapy need to be comprehensively assessed and managed in combination with the tumor type and preconditioning regimen.

#### 2.2.3 Pancreatic toxicity

Elevated lipase or amylase levels have been observed in CAR-T therapies targeting EGFR and CLDN18.3 (66). In a CAR-T cell therapy study for NSCLC, a case of transient Grade 3-4 lipase elevation was reported. The specific causes of pancreatic toxicity in these studies are unclear, and a direct correlation between the elevation and abdominal pain symptoms has not been established (85). Since epithelial cell adhesion molecule (EpCAM) is overexpressed in various tumors as well as tissues such as the pancreas, CAR-T therapy targeting EpCAM theoretically may induce pancreatitis, requiring particular vigilance (86).

#### 2.2.4 Hepatic toxicity

Hepatic toxicity is not prominent in CAR-T therapy for solid tumors (87), and is mainly detected through laboratory tests, such as elevations in transaminases, bilirubin, gamma-glutamyltransferase, and alkaline phosphatase (17, 88, 89). Mild elevations in liver enzymes do not necessarily lead to changes in liver tissue structure (78). When hepatic toxicity due to CAR-T is suspected, it needs to be differentiated from elevations in liver enzymes caused by liver metastasis (90).

#### 2.2.5 Lung toxicity

CAR-T therapy can induce immune-related lung toxicity manifesting as cough, dyspnea, and other symptoms, severe cases may lead to pulmonary edema and ARDS (91), which may be related to cytokine release and off-target effects. Fatal cases have been reported in treatments targeting EGFRvIII and ERBB2 (92, 93). CAR-T cells targeting CEACAM5 drive localized lung toxicity, although the lung toxicity is transient and no patients required invasive ventilation. Despite prophylactic antibiotics, no respiratory tract infections were confirmed, and no systemic steroids were required. Immunomodulation may reduce toxicity but also decrease the efficacy of CAR-T therapy (42). High-resolution CT is the preferred diagnostic tool when lung toxicity is suspected (94–96), and severe cases may require mechanical ventilation and IL-6 inhibitor therapy. To mitigate off-target effects, researchers have developed CAR-T cells targeting CDH17, which have shown good antitumor efficacy and no toxic side effects in mouse models (97).

#### 2.2.6 Renal toxicity

Renal toxicity mainly manifests as acute kidney injury (AKI), with CRS being a major contributing factor. The severity is graded based on the degree of serum creatinine elevation or the need for renal replacement therapy (RRT). Some patients experience AKI after CAR-T therapy, with a minority requiring RRT, and individual cases have resulted in death during severe CRS (98).

#### 2.2.7 Mucocutaneous toxicity

Dermatological adverse events (DAEs) related to CAR-T therapy are rarely reported, but severe rash and vascular skin reactions are associated with high mortality. The median time of DAE onset is 3 days after CAR T-cell infusion (99). In CAR-T therapy targeting EGFR, skin toxicity manifests as oral mucositis, oral ulcers, etc., mostly grade 1-2, with a minority reaching grade 3-4 (100). Severe skin toxicity can be alleviated with treatment using corticosteroids, intravenous immune globulin, and etanercept (101, 102). Skin toxicity may be related to CAR T-cells attacking target antigens expressed on normal epithelial cells and vascular endothelial cells.

### 2.3 Long-term adverse effects related to the combined action of multiple mechanisms antifungal drugs as tumor immunotherapy sensitizers

Despite the significant short-term efficacy demonstrated by CAR-T cell therapy, its long-term adverse effects remain an issue of concern. With the widespread clinical application of CAR-T cell therapy, its long-term adverse effects have gradually emerged. These long-term adverse effects not only affect the quality of life of patients but may also pose a threat to their safety (23). The long-term adverse effects of CAR-T cell therapy mainly include cytokine-associated hematopoietic toxicity (CAHT) and secondary malignancies (23).

#### 2.3.1 Cell-associated haematotoxicity

Although CAR-T cell therapy is effective, the common adverse effect of CAHT impacts patients’ immune function (39), increasing the risk of infection and bleeding (103, 104). This condition varies based on the treatment target, CAR-T type, and patient differences, with symptoms including anemia, leukopenia, and thrombocytopenia (105). It has been confirmed that most infectious events occur within 30 days after CAR-T cell infusion, with bacterial infections being dominant, mainly including bloodstream infections and respiratory infections (106). A small proportion of patients also experience infections between Day 31 and Day 180 after anti-CD19 CAR-T treatment (107).

The mechanisms involved include suppression of bone marrow hematopoiesis, cytokine release, and co-expression of target antigens (108). Clinical practice should involve close monitoring of blood parameters, using the CAR-HEMATOTOX score to identify high-risk individuals (105), and targeted treatment such as blood transfusions, antibiotics to prevent infection, and avoiding drugs that increase bleeding risk (109, 110). The CAR HEMATOTOX score is a specific rating system designed to assess the risk of hematological toxicity in patients undergoing CAR-T cell therapy (111). It integrates multiple clinical and laboratory parameters, such as blood cell counts, biochemical indicators, and other relevant factors during the treatment process, to comprehensively evaluate the likelihood of patients developing severe hematological toxicity (112). For B/T cell aplasia and infection, attention should be paid to immune status and infection prevention and control (113). Special vigilance is required for encephalitis caused by HHV-6 reactivation, and HHV-6 screening is necessary before treatment (114). In the early stages of CAR-T therapy, prophylactic anti-infective drugs should be promptly administered (115–119). The utility of Procalcitonin (PCT) in risk stratification and diagnosis of infectious complications in high-risk patients after CAR-T cell therapy is continued, with a PCT threshold of 1.5μg/L advocated for the diagnosis of sepsis. In situations where white blood cell count and CRP values are unreliable, particularly during CRS and lymphocyte depletion, a PCT value < 0.5 μg/L may help exclude sepsis (120).

#### 2.3.2 Secondary primary malignancies

Due to impaired immune function and decreased immunoglobulin levels, patients are at increased risk of infection (121). Studies have found that when the median follow-up time reaches 3 years, approximately 6.5% of patients with hematological malignancies develop SPMs. The cause of death in these patients may be related to immunosuppression in patients undergoing CAR-T cell therapy, who may have underlying diseases, have previously undergone cancer treatment, or have received immunosuppressive chemotherapy as lymphocyte-depleting therapy (122), rather than due to erroneous insertion of chimeric antigen receptor genes during cellular gene engineering (123). Another perspective is that the occurrence of these malignancies may be related to the attack of CAR-T cells on normal cells or to gene instability induced by CAR-T cells (124). The long-term presence and activation of CAR-T cells in the body suggest that tolerable long-term adverse effects may indicate a better prognosis for patients (125, 126). The working mechanism, kinetic characteristics, persistence, factors related to prognosis, and potential side effects are among the multiple aspects involved in CAR-T cell therapy. CAR-T cells are living drugs with proliferative capacity and unique cellular kinetic characteristics. Over time, the number of CAR-T cells decreases, partly due to activation-induced cell death (127, 128). However, a small subset of CAR-T cells can maintain a memory cell phenotype for months to years, generating sustained antitumor activity. Persistence is one of the key indicators for assessing the effectiveness of CAR-T cell therapy (129, 130). Long-term follow-up data indicate that CD19 CAR-T cells can induce long-term remission in patients with B-cell malignancies, often with minimal long-term toxicity, and potentially lead to cure in some patients (125, 131). Factors associated with durable remission after CAR-T cell treatment include deep initial remission, lower baseline tumor volume, absence of extramedullary disease, higher peak levels of circulating CAR-T cells, and the administration of lymphodepleting chemotherapy (132, 133). The long-term presence and activation of CAR-T cells in the body continuously exert antitumor effects, thereby extending patients’ survival (134). However, it is inevitable that CAR-T cells may also cause damage to normal cells and even induce gene instability (124, 135), leading to immunosuppression and impaired immune function.

Research indicates that gene integration via viral vectors may trigger insertional oncogenesis, which raises significant concerns for gene therapy. Historical gene therapy trials have documented cases of leukemia arising from the inactivation of tumor suppressor genes, such as LMO2, due to integration events, highlighting the necessity for careful selection of integration sites (136–138). Recent clinical trial data show a decrease in adverse events related to integration; however, ongoing monitoring of T cell clonal expansion and long-term stability is essential. Although follow-up results suggest that vector integration does not significantly increase the incidence of SPMs researchers remain cautious about potential risks related to clonal expansion in certain patients (124, 139, 140). This underscores that, while current T cell therapies generally exhibit high safety, there is still a need for more precise vector designs to mitigate risks.

The occurrence of secondary primary malignancies is a critical safety concern in T cell therapies. The study conducted long-term observations on 783 patients who underwent CAR-T cell therapy and found that the overall incidence of secondary primary malignancies (SPMs) was 2.3% (141). Many affected patients have a prior history of malignancies or a high-risk genetic background. Although some cases may be linked to viral vector integration, a clear causal relationship has not been established. The remaining SPM cases involved solid tumors predominantly located in the liver, stomach, and lungs, likely connected to underlying health issues or chronic immunosuppression. Further analysis of integration site data revealed no direct association with oncogenes. Although most secondary primary malignancies (SPMs) occur within five years after treatment, there was one case of papillary thyroid cancer as an SPM that emerged 14 years post-treatment. However, it is noteworthy that the majority of SPMs actually occur within 1-3 years after treatment, which is a critical period when virus vector integration may pose risks. Follow-up data further indicates that the risk of new SPMs decreases over time, suggesting that the long-term impact of virus vector integration may be relatively minor (141).

CAR-T cell therapy aims to combat malignant tumors by reprogramming the patient’s immune system, but this process can come with significant downsides. For some patients, the treatment may lead to various immune suppression issues. Research has identified two primary manifestations of this immunosuppression: the depletion of normal B cells and immune remodeling following CRS (142). CAR-T therapy targets cells expressing the CD19 antigen, but because normal B cells also express CD19, patients may experience considerable B cell depletion, known as B-cell aplasia, which can impair antibody production and persist for years. Additionally, patients who undergo severe CRS may require an extended period for their immune systems to recover. During this recovery phase, their ability to fend off infections is notably diminished, often accompanied by prolonged inflammation and disruptions in immune function. This mechanism might also increase the susceptibility of SPM. This should be considered seriously for trials and attempts to introduce CAR T cells earlier in treatment, where the risks might be more concerning compared to other established therapies (143).

## 3 Analysis and prospects

The application of CAR-T therapy in solid tumors poses greater challenges due to the complexity of the tumor microenvironment and a series of adverse reactions induced by the therapy. In this review, we comprehensively analyzed the potential mechanisms of adverse reactions to CAR-T cell therapy in solid tumors and classified them into three major categories: adverse reactions associated with high levels of proinflammatory cytokines, adverse reactions caused by shared antigens between tumors and healthy tissues, and long-term adverse reactions resulting from a combination of multiple mechanisms.

CRS and CANS are typical examples of adverse reactions triggered by elevated cytokine levels. Off-target effects resulting from shared antigens between tumors and healthy tissues lead to organ toxicity, exemplified by reported cardiovascular, gastrointestinal, pancreatic, hepatic, lung, renal, and mucocutaneous toxicities. The severity of these toxicities is often correlated with the extent of CRS, thus, it is crucial to have a thorough understanding of the patient’s tumor antigen profile and to carefully select CAR-T targets to minimize off-target effects.

To overcome these challenges, various strategies need to be explored, such as improving the infiltration ability of CAR-T cells (144), enhancing the survival and function of CAR-T cells (145), utilizing CAR-T cells that target the tumor microenvironment (146), adopting combined treatment strategies, and optimizing the therapeutic regimen of CAR-T cells (147).

CAR-T therapy is accompanied by a series of long-term adverse reactions, with CAHT and secondary malignancies standing out as particularly notable. These complex reactions further complicate the treatment process. These long-term impacts underscore the urgent need for continuous monitoring of patients and the adoption of proactive management strategies to ensure their safety and quality of life are adequately protected. Looking ahead, research efforts can focus on several key directions: Firstly, delving deeper into the underlying mechanisms and clinical manifestations of long-term adverse reactions associated with CAR-T cell therapy, aiming to provide a solid theoretical foundation for clinical diagnosis and treatment. Secondly, striving to develop innovative CAR-T cell design strategies to reduce their potential attack on normal cells, thereby effectively minimizing off-target effects and the incidence of long-term adverse reactions. Furthermore, exploring new avenues for the combined application of CAR-T cell therapy with immunomodulatory agents, with the aim of reducing the intensity of immune responses and alleviating long-term adverse reactions. Lastly, strengthening long-term follow-up and monitoring mechanisms for patients to ensure that any long-term adverse reactions are promptly identified and appropriately managed, thereby comprehensively enhancing patients’ quality of life and treatment outcomes.

In summary, despite the promising prospects of CAR-T cell therapy in treating solid tumors, its clinical application must be cautiously conducted based on a thorough understanding of potential adverse reactions and their underlying mechanisms. Future research should focus on improving CAR-T design to enhance specificity and reduce off-target effects, developing more effective and safer strategies for managing CRS and CANS, and exploring long-term monitoring programs to identify and mitigate the risks of CAHT and secondary malignancies.
